# Precision treatment in breast cancer: Leveraging genetic interactions

**DOI:** 10.1002/ctm2.70407

**Published:** 2025-07-23

**Authors:** Cai‐Jin Lin, Xin Hu, Zhi‐Ming Shao, Yi‐Zhou Jiang

**Affiliations:** ^1^ Departments of Breast Surgery and Oncology, Key Laboratory of Breast Cancer Shanghai Medical College Fudan University Shanghai Cancer Center Fudan University Shanghai China; ^2^ Precision Cancer Medical Center Fudan University Shanghai Cancer Center Shanghai China

## 1 CHALLENGES IN PRECISION TREATMENT BASED ON INDIVIDUAL GENOMIC ALTERATIONS

**FIGURE 1:**
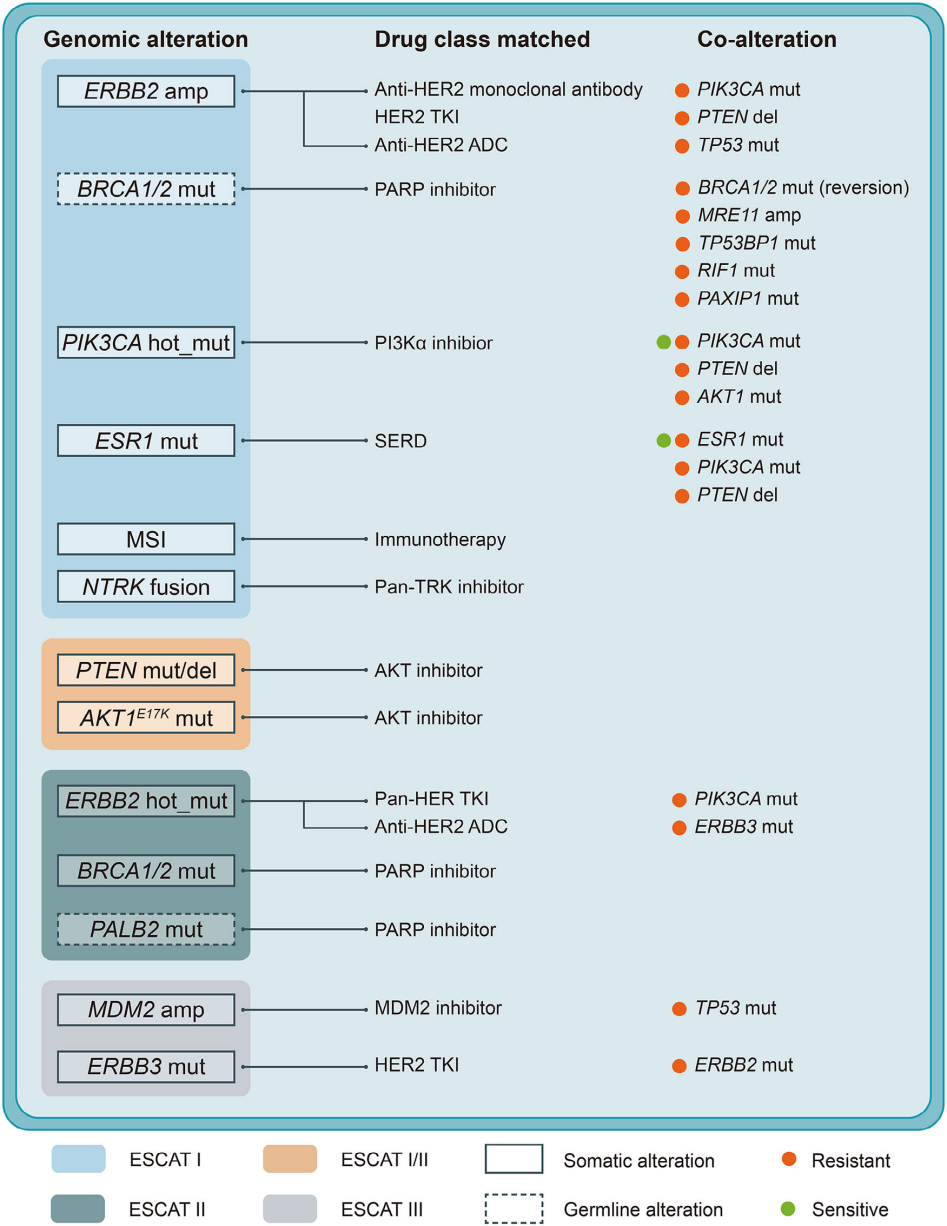
Genomic alterations with clinical actionability in breast cancer precision oncology. ADC, antibody‐drug conjugate; hot_mut, hotspot mutations; SERD, selective estrogen receptor degrader; TKI, tyrosine kinase inhibitor; TRK, tropomyosin receptor kinase.

The advent of next‐generation sequencing technology has catalyzed significant advancements in precision treatment strategies for breast cancer by leveraging insights into individual tumour genomes.^1^, ^2^ This genome‐guided approach has notably enhanced therapeutic outcomes, particularly in patients with specific genomic alterations (Figure 1).^3^ For example, anti‐HER2 targeted therapies exemplify a classical form of genotype‐matched treatment specifically designed to target gene products in *ERBB2*‐amplified breast cancers.^4^, ^5^ However, despite the initial success of precision treatment in breast cancer, clinical endeavours targeting individual alterations have yielded variable efficacy. One important reason is that clinical decision‐making for precision treatment has overly focused on single driver alterations without considering potential interactions among multiple genomic alterations and their impact on efficacy.^6^

## 2 GENETIC INTERACTIONS REFINE PRECISION TREATMENT STRATEGY BEYOND INDIVIDUAL ALTERATIONS

The concept of genetic interactions, encompassing both the co‐occurrence and mutual exclusivity of genomic alterations, highlights the complexity underlying breast cancer biology and treatment response.^7^ Specifically, co‐occurring genomic alterations can synergistically impact tumour behaviour and therapeutic responses (Figure 1). Targeting only one genomic alteration may lead to the development of drug resistance, whereas simultaneous targeting of both events has the potential to effectively eradicate the tumour. For instance, the presence of *PIK3CA* co‐mutations has been associated with poorer prognosis under anti‐HER2 therapy.^6^ In addition, interactions such as *HER2‐HER3* co‐alteration influencing neratinib response via PI3K activation illustrate potential mechanisms of treatment resistance that can be overcome through a combination therapy of neratinib and PI3Kα inhibitors.^8^ Despite some existing insights into mutation‐treatment interactions,^9^ comprehensive investigations into the broader landscape of genetic interactions and their implications for treatment outcomes remain crucial.

These challenges necessitate a comprehensive investigation of genetic interactions, especially co‐occurrences, and their association with treatment outcomes in breast cancer. Accordingly, we developed a comprehensive network of genetic interactions specific to breast cancer, identifying 50 instances of co‐occurrence and 30 instances of mutual exclusivity.^10^ This network not only reaffirms known associations, such as the co‐occurring *TP53* mutation and *MYC* copy number amplification, as well as mutually exclusive mutations between *PIK3CA* and *AKT1*, but also unveils novel genetic interactions, such as the co‐occurring *TP53* mutation and *MYB* copy number amplification, previously unreported in the literature. Subsequent analysis of these interactions in prospective clinical cohorts revealed their prognostic and predictive values, informing potential therapeutic strategies. For genetic interactions with significant translational value, we employed various experimental models, including cell lines, patient‐derived organoids, tumour fragments, and in vivo systems, to elucidate their functional roles and underlying molecular mechanisms. Notably, the co‐occurrence of *TP53* mutation and *AURKA* copy number amplification is observed in approximately 4.9% of hormone receptor‐positive/HER2‐negative (HR+/HER2‐) breast cancer cases. This co‐alteration contributes to tamoxifen resistance by promoting centrosome amplification and aneuploidy. The co‐occurrence of germline *BRCA1* mutation and *MYC* copy number amplification is present in about 4.6% of triple‐negative breast cancer (TNBC) cases, sensitizing TNBC to PARP inhibitors by inducing DNA double‐strand breaks and genomic instability. Furthermore, the co‐occurring *TP53* mutation and *MYB* copy number amplification, found in approximately 7.3% of TNBC cases, are associated with resistance to immunotherapy due to compromised neoantigen presentation. These findings, together with existing evidence, should be taken into account when determining therapeutic strategies.

Previous studies have performed systematic analyses of co‐occurrences and mutual exclusivities in pan‐cancer cohorts.^7^ However, despite identifying novel genetic interactions through our comprehensive multi‐omics data, the central focus of our study lies in elucidating genome‐informed treatment decisions behind these co‐alterations. The clinical relevance of such co‐alterations has not been comprehensively studied in prior research. Indeed, we have identified several novel co‐alterations with potential implications for enhancing therapeutic effectiveness in clinical settings. More importantly, our study also provides insights into the relationship between these co‐occurrences and their corresponding biological properties and potential mechanisms that may underlie treatment resistance or sensitization in patients. Consequently, we believe our study constitutes more than a mere additive contribution to the existing studies; rather, it is an in‐depth study providing novel biological findings and translational values.

## 3 FUTURE OUTLOOK

Moving forward, while our study investigated genetic interactions and characterized their biological properties and therapeutic potential based on a large‐scale multi‐omics cohort and a real‐world clinical sequencing cohort, the field is advancing with new cohorts profiled in a comprehensive and multi‐modal manner using cutting‐edge technologies. For instance, single‐cell DNA sequencing holds promise for deeper insights into intra‐tumoural heterogeneity and tracing clonal evolution, which are critical for understanding genetic interactions at finer resolutions. In addition, it is essential to expand investigations to encompass interactions among multiple genomic alterations or between genomic and transcriptomic alterations. Moreover, while our study primarily focuses on protein‐changing alterations, future research should also explore interactions involving silent alterations to comprehensively capture the genomic landscape's impact on treatment efficacy.

In conclusion, as precision treatment continues to evolve, incorporating insights from genetic interactions promises to refine therapeutic strategies, offering personalized approaches that maximize efficacy and overcome resistance mechanisms in breast cancer treatment.

## AUTHOR CONTRIBUTIONS

C.J.L., X.H., Z.M.S., and Y.Z.J. contributed to literature review and manuscript writing.

## CONFLICT OF INTEREST STATEMENT

The authors declare no conflict of interest.

## CONFLICT OF INTEREST STATEMENT

Not applicable.
